# The Architecture of Connectivity: A Key to Network Vulnerability, Complexity and Resilience

**DOI:** 10.1007/s11067-022-09563-y

**Published:** 2022-05-17

**Authors:** Aura Reggiani

**Affiliations:** Department of Economics, University of Bologna, Piazza Scaravilli 2, 40126 Bologna, Italy

**Keywords:** Connectivity Architecture, Network Vulnerability, Complexity, Network Resilience, Spatial and Transport Economics

## Abstract

This paper highlights the relevance of connectivity and its architecture as a general conceptual framework which underlies and integrates the concepts of network vulnerability, complexity, and resilience. In particular, it will be pointed out that connectivity architecture can be considered an explicit key element for network vulnerability and shock propagation. While the relevance of the various connectivity configurations is not clearly emphasised in the dynamic complexity models of the space-economy, it appears to play a primary role in network analysis. In this regard, the emerging recognition of connectivity architecture in relation to hubs ‒ and hierarchies of hubs ‒ in a complex network will help the enhancement of network resilience. The paper develops as follows. First, the notion of network vulnerability, which refers not only to the phenomenon of shocks, but also to the propagation of shocks in a network, will be examined. Here it appears that modelling vulnerability and shock propagation, also jointly with cascading disaster models, is strongly based on connectivity issues. The question is: How can conventional (complex) system dynamic modelling, as well as network modelling, take into account these shocks and connectivity dynamics from the methodological viewpoint? A review in this respect shows how connectivity is a ‘hidden’ element in these complexity models, for example, in chaos or (dynamic) competition models, where interaction parameter values might lead to vulnerable domains and chaotic behaviour. On the contrary, connectivity and its various topologies have a distinct, primary role in network analysis. The issue of network resilience appears therefore to be the ‘response’ to vulnerability and chaos, calling for robustness and stability of the network in the presence of shocks and disruptions. Resilience analysis refers to the speed at which a network returns to its equilibrium after a shock, as well as to the perturbations/shocks that can be absorbed before the network is induced into some other equilibrium (adaptivity). Connectivity is relevant here, but not often considered in spatial economics. In order to reach a unified methodological framework, attention will finally be paid to a complementary analysis of the (dynamic) concepts of vulnerability and resilience. In this light, chaos models/properties might be seen in a positive perspective, since small changes can lead to uncertain and unstable effects, but also, thanks to connectivity, to new equilibria which are not necessarily negative. Thus, the architecture of connectivity, in its interdisciplinary insights, can be considered as a fundamental (and analytical) approach for identifying vulnerability and resilience patterns in complex networks.

## Background

Natural and man-made disasters, economic recession and financial dynamics, geo-political changes, ruptures, and collapses in the infrastructural and communication networks, terrorist attacks, and sudden critical events of high impact, such as the current contagious diseases due to Covid-19, are not only causing enormous socio-economic damage but also creating an era of uncertainty, which is leading researchers and scientists to focus on concepts and themes such as the vulnerability, and hence the complexity and, hopefully, the resilience, of spatial economic networks.

In this context, the concept of connectivity, closely linked to vulnerability, complexity, and resilience, needs particular attention.

First, connectivity has been highlighted as complex (network) connectivity in the context of globalisation studies. For example, Tomlinson refers to globalisation as: ‘*an empirical condition of the modern world: what I shall call complex connectivity. By this I mean that globalisation refers to the rapidly developing and ever-densening network of interconnections and interdependencies that characterizes modern social life*’ (1999, p. 2). In his discussion, Tomlinson also emphasises that: ‘*the broad task of globalization theory is to understand the sources of this condition of complex connectivity and to interpret its implications across the various spheres of social existe*nce’ (1999, p. 3).

Along these lines, connectivity has been firmly embedded in past studies and in different disciplines, such as physics, sociology, politics, economics, etc., where the network concept came to the fore for its important features: ‘*The modern spatial economy has a global ‘networked’ character that is generating important socio-economic and political changes. In this respect, new forms of connectivity play a significant role through their dynamic and complex interplay with the economic and political driving forces behind globalization*’ (Reggiani 2021, p. 325). In particular, in recent years, connectivity has been considered mainly with reference to transport, telecommunication, and social networks, where the role of network connectivity is tangible (Caldarelli and Vespignani 2007; Reggiani 2012).

It should be noted that in 1962 the Nobel Laureate in Economics, Herbert Simon, wrote an article entitled “The Architecture of Complexity”, where he emphasised the role of hierarchical structures in complex systems: ‘*Thus my central theme that runs through my remarks is that complexity frequently takes the form of hierarchy*, *and that hierarchic systems have some common properties that are independent of their specific content. Hierarchy, I shall argue, is one of the central structural schemes that the architect of complexity uses*’ (Simon 1962, p. 468). Not surprisingly, 45 years later the physicist Albert-László Barabási wrote an article with the same title “The Architecture of Complexity”, where he underlined, first, the role of networks intrinsically linked to complex systems: ‘*Rather, in complex systems, the interactions form networks, where each node interacts with only a small number of selected partners whose presence and effects might be felt by far away nodes*’ (Barabási 2007, p. 33); and second, the relevance of hierarchical structures: ‘…*a thorough understanding of complex systems requires an understanding of network dynamics as well as network topology and architecture*’ (p. 34).

So, both these scientists, although from different disciplines, stressed how the study of a complex system should consider not only the nature of dynamical processes, but also its network architecture, in other words its interacting connectivity structure. In this vein, it should be noted the term ‘system’ used in this paper embeds the network concept.

Inspired by these two works, in the present paper we go a step further, by considering a ‘nested approach', i.e., by paying attention not only to connectivity, but also to the architecture of connectivity for vulnerability and resilience issues. More specifically: if we interpret connectivity as the (weighted) degree of network linkage of a given node in a network, its architecture also has a relevant role for both vulnerability and resilience.

In parallel to complex networks, the concepts of vulnerability and resilience have been widely investigated in recent years. Nevertheless, their importance is still increasing as they are often being applied in economics, in transport, urban and regional studies, and, more generally, in the social sciences. Vulnerability analysis essentially refers to the propagation of shocks in a network, while resilience analysis refers to the speed at which a network returns to its equilibrium after a shock, as well as to the perturbations/shocks that can be absorbed before reaching new equilibria. Thus, vulnerability and resilience are clearly related to the complexity of network evolution in the presence of shocks. However, connectivity and its impact on network vulnerability and resilience has not yet received ‘full’ attention, despite the huge number of studies on vulnerability and resilience.

Given this background, the present paper highlights methodological considerations on the role and interpretation of connectivity – and its architecture – in vulnerability and resilience in complex networks. Attention will also be paid to a complementary analysis of these two (dynamic) concepts.

In sum, on the basis of the existing literature, it will be argued that connectivity and its architecture can be considered as a useful (and analytical) framework for understanding and interpreting the concepts of network vulnerability and resilience.

In this regard, we can distinguish the following aspects of connectivity, which will be discussed in the course of the paper:

- *Explicit connectivity:* network vulnerability and shock propagation.
- *Hidden connectivity:* complexity in the space-economy.
- *Relevance of connectivity*: complexity in network analysis.
- *The architecture of connectivity*: resilience in spatial and transport economics.

Sections 2, 3, 4, and 5 will, in turn, explain these four aspects – and the related scientific challenges – in the light of a possible integrated approach. Finally, Sect. 6 concludes with some considerations on the need for an interdisciplinary perspective provided by complexity science.

## Explicit Connectivity: Network Vulnerability and Shock Propagation

### Network Vulnerability

Vulnerability is generally identified as a characteristic or a property of dynamic networks: in contrast to resilience, it has a negative connotation, as it refers to the overall reduction of a network’s quality as a consequence of the action of factors which stress the system (Reggiani et al. 2015). ‘*For this reason, vulnerability may be interpreted as the other side of the coin with respect to resilience*’ (Reggiani et al. 2015, p. 7).

In particular, vulnerability is about the susceptibility of the (socio-economic) system or *any of its constituents* to harmful external pressures (Seeliger and Turok 2013). According to this definition, the vulnerability of the system’s (individual) components is also relevant: as a result, the architecture/structural configuration of the system in the presence of the shock seems to play a critical role.

It should be noted that vulnerability is an issue that is rarely treated in spatial economics, while it is analysed in detail in transport economics. Studies on transport vulnerability are increasing (Mattsson and Jenelius 2015). Following the above definition of Seeliger and Turok, transport vulnerability can be assumed to be ‘*the susceptibility of systems to extreme tensions (incidents, disturbances, disruptions, *etc*.) with a reduction in its service level*’ (Gonçalves and Ribeiro 2020, p. 7). For example, in a physical infrastructure network, the rupture of a link immediately triggers a change in the performance of the whole network and alternative transport modes. However, a formal and commonly accepted definition of network vulnerability is still lacking as highlighted, in the past, by several authors (Berdica 2002; Husdal 2004; Taylor and D’Este 2007), as well as in more recent years, in review studies on transport vulnerability (e.g., Gonçalves and Ribeiro 2020; Reggiani et al. 2015), and in studies of vulnerability in the road network (e.g., Watling and Balijepalli 2012) and the rail network (e.g., Neves et al. 2021).

What most authors do agree on is that vulnerability should focus on the impacts of the different threats to the network, by posing the following questions (Husdal 2004):

- “Vulnerable…where?”, in order to identify the location of the impacts within the network.
- “Vulnerable…to what?”, in order to identify the particular circumstances or external threats and their probable occurrences.
- “Vulnerable…how?”, in order to address the particular scenario and its impact and how the network may or may not cope with it.

However, even extreme conditions may not necessarily lead to a full breakdown of the network, if the network is sufficiently equipped to handle such situations or events. From here the link with resilience emerges, i.e., the network’s ability to deal with shocks and to adapt to their consequences (see also Sect. 5).

Reggiani et al. (2015), in their review, highlight a range of various meanings and descriptive categories linked to the vulnerability concept, such as reliability, variability, or fragility: ‘*All such concepts are employed to map out the features of a transitional movement of a transport system that is affected by a shock*’ (Reggiani et al. 2015, p. 13). In this context, it is worth recalling that transportation systems have a (complex) network character: therefore, it is plausible that the transport vulnerability properties are related to a core network concept, viz. connectivity.

Connectivity, which can be defined as the ease of two or multiple nodes to interact, seems to be a *necessary condition* for network vulnerability. If we look at the measurements of vulnerability in the transport literature, we observe the relevance of connectivity by means of analyses of multiple links/edges failure, link-based indicators, and dynamic networks in continuous and discrete time (Annex B in Reggiani et al. 2015; Yap et al. 2018). In addition, the specific definition of transport vulnerability as *reduced accessibility* proposed by Berdica (2002), and consolidated by other authors (e.g., Kondo et al. 2012; Taylor et al. 2006), has paved the way for empirical analyses on the relationship between accessibility, as an analytical tool embedding connectivity, and vulnerability/resilience (Östh et al. 2015).

In sum: connectivity is an explicit factor which impacts network vulnerability.

Closely linked to the concept of vulnerability is the concept of resilience (Sect. 5). In general, several authors consider vulnerability to be a pre-event characteristic, and resilience to be the outcome of a post-disaster response, i.e., resilience as the responsiveness of the system to a shock (Adger 2000; Cutter et al. 2008; Foster 2007; Pendall et al. 2012; Rose 2007). Thus, resilience is often seen as a way to reduce vulnerability, and a more resilient system is a system with less vulnerable sub-systems (Pendall et al. 2012).

A further element to be considered – in this analysis on network vulnerability – is the relevance of shock propagation, since not only the (local) network structure but also the propagation mechanism influences the dynamics of propagation, by identifying various vulnerability patterns.

### Shock Propagation

The concept of vulnerability may provide new elements for the study of dynamic behaviour, since it gives the opportunity to explore not only multiple network (dis)equilibria in the presence of a shock, but also shock propagation mechanisms within the network itself.

Starting from the seminal works of Frisch (1933), Simon and Ando (1961), and Ando and Fisher (1963), propagation and its dynamics have been studied in the past in a vast range of contexts. We may recall contributions which analyse, among other things: disease effects (Dodds and Watts 2004); social contagion (Burt 1987); social influence (Turner 1991); social capital (Coleman 1998); epidemiology (Rahmandad and Sterman 2008); the diffusion of innovation (Rogers 1995); information diffusion (Dodds et al. 2003; Reagans and McEvily 2003); the production process (Hagemann and Scazzieri 2009; Landesmann and Scazzieri 1990) (for a review, see Vermeer 2015). This stream of work aims to understand the dynamic propagation processes and their outcomes.

Interestingly, already in 1933, Frisch distinguished between the propagation problem (i.e., the structural properties of the swinging system) and the exterior impulse problem, by highlighting the necessity to develop dynamic theory in this regard. More recently, Borgatti (2005) advocated that shock propagation, considered as a ‘flow’ in a network, can be decomposed into three dimensions: depth, strength, and width. In particular: depth indicates how far propagation will reach the nodes; strength identifies the tendency of a shock to amplify/dampen; and width reflects the different directions of propagation in the network (Vermeer 2015). These three dimensions are captured by three sub-processes: radiation, transmission, and reception. Among these sub-processes, recent works have paid a great deal of attention, with respect to transport and migration networks, to radiation process and related models (Simini et al. 2012; Varga et al. 2016), as an alternative theoretical framework to the well-established spatial interaction/entropy models (Wilson 1970). In this context, investigation into the role of different types of shock would also be interesting.

In view of this, the propagation mechanisms, together with conventional dynamic models, seem worth exploring, in order to capture agents’ dynamic behaviour. For example, clusters that capture agents/entities which show a high propensity to propagate can act as hubs in a complex network (Jackson and Lopez-Pintado 2013). Along these lines, it is interesting to mention cascading disaster models, which capture all three dimensions of propagation, but also to consider *the size of cascade* to be an important characteristic of the cascade (Buzna et al. 2006; Vermeer 2015). Hence, large cascades of influence are driven not only by influential (hubs), but also by a critical mass of easily influenced individuals/nodes (Watts and Dodds 2007).

In other words, in order for a cascade to spread globally, the population must contain a *connected network* of *vulnerable* elements/individuals/nodes, i.e., a ‘critical mass’ that ‘percolates’, in the sense that it ‘reaches’ the population (Stauffer and Aharony 1992; Watts and Dodds 2007).

To sum up, from the above considerations on vulnerability analysis, connectivity appears to be a key element in generating and explaining network vulnerability.

The emerging research question is then: Whether and how has the relationship between shocks and connectivity been taken into account, in the past, in the dynamic modelling of (complex) spatial economic systems, especially in the light of vulnerability/resilience issues?

## Hidden Connectivity: Complexity in the Space-Economy

### Complexity and Connectivity

In the past decade, complexity has become an important and fascinating domain for advanced research on nonlinear dynamics, in which a multiplicity of scientific fields are involved (economics, social sciences, life sciences, geography, physics, and so forth). Complex systems analysis refers to research at the dynamic interface of – or the interaction between – the micro or meso-elements of a system that are interconnected and determine a macro-level of operation of the system that is not just the sum of the system’s individual elements (Casti 1979; Simon 1962).

The term complexity derives from the Latin word ‘complexus’ which means ‘*entwined*’, ‘*twisted togethe*r’. In addition, the Oxford Dictionary defines ‘complex’ as ‘*made of many different things or parts that are connected*.’ Thus, connectivity seems to be closely related to the complexity concept. Casti (1979) introduces a distinction between ‘static complexity’ and ‘dynamic complexity’, which involves the distinction between static and dynamic connectivity:

- *Static complexity* refers to the network configuration, where the components are put together in an interrelated and intricate way, as measured, for example, by the number/type of hierarchical structures and connectivity patterns, the variety of components, and the strength of interactions within the system.
- *Dynamic complexity* concerns the dynamic network behaviour governed by nonlinearities in the interacting components and measured by computational and evolutionary complexity models (Casti 1979; Reggiani 2021).

It should be noted that, amongst the numerous definitions of complexity, the above definition by Casti (1979) might be associated with the classification of complexity in terms of: a) structural measures; and b) dynamic measures, proposed by Wackerbauer et al. (1994).

Therefore, the term ‘complexity’ embeds both the assemblage of different units in a system and their intertwined dynamics, by means of connectivity patterns. In other words, the term ‘complexity’ *embeds the concept of network* (net-works = ‘operations via nets’) (see also Reggiani 2021). The most important element here is the (random) dynamic behaviour of the complex system, which is difficult to predict.

At the same time, complex systems (networks) have an underlying architecture guided by universal principles, including the dynamic properties of hierarchically organised systems. In other words, there are hierarchies and stratifications in complex dynamic systems that greatly simplify their behaviour (Scazzieri 2021; Simon 1962).

A further methodological step is then the relevance of the *simplicity concept*, which appears to be intrinsically related to the concept of complexity since it seems to be the only way of ‘governing’ complexity (Reggiani and Nijkamp 2009). In this vein, harnessing complexity (Axelrod and Cohen 2000) involves the need to provide a device for channelling complexity. We then see very simple models displaying static and dynamic complexity in spatial economics. A review of these models is in Reggiani (2021). Here we recall only the most important dynamic models, in the light of the related connectivity features.

### Dynamic Complexity and Vulnerability in Spatial Economics

The complexity movement has also had far-reaching impacts on dynamics research in spatial economics. The space-economy is often interpreted as a standard well-functioning economic system enriched with the element of space. But space is not just an additional dimension of the economy: it forms an intrinsic feature of any socio-economic system and may lead to the emergence of complex nonlinear and interactive behaviours and processes in a geographical setting.

The foundation for an interpretation of the space-economy as an interdependent complex set of economic relationships – at different geographical scales and with a variety of time dimensions involved – can be found in the ‘first law of geography’ formulated by Tobler (1970), who stipulates that everything in space is related to everything else, but near things are more related than distant things. In this vein, spatial interaction models, borrowed from physics and interpreting Newton’s law in statistical-economic terms, demonstrate the simplicity of modelling large-scale networks (Reggiani 2021; Reggiani et al. 2021). The solidity of these laws can then be reconsidered in the light of recent advances in complexity theory: connectivity, even though not explicitly considered in these spatial economic models, appears to exert a relevant impact on an organised space.

One of the striking features in the modern space-economy has been the simultaneous occurrence of spatial dynamics (both fast and slow dynamics) and spatial inertia (e.g., persistent welfare disparities between regions). Regions and cities are apparently operating in a complex force field, with asynchronously emerging key factors that impact on regional or urban development in different ways and with different rates of growth^1^ (see also Batty 2005, 2013).

Given the objective of ‘capturing’ complexity in association with its stability/instability characteristics, the (hidden) role of the architecture of connectivity in this regard can be highlighted in simple and universal dynamic models, as subsequently described.

As a result of self-organising forces among interacting micro- or meso -units, a dynamic network configuration may emerge that displays its own dynamics, ranging from stability to cycles and to unstable/irregular effects caused by bifurcations with unexpected phase transitions. In particular, chaos theory, dealing with deterministic non-linear systems which are able to produce complex motions of such a nature that they seem completely random, can show vulnerability features associated with irregular/unpredictable behaviour. A prototype example of a chaos model is May’s model (in one dimension) which reads as follows (Gandolfo 1996; May 1976; Wackerbauer et al. 1994):

1 $${x}_{t+1}=r {x}_{t}\left(1-{x}_{t}\right)\qquad x\ \epsilon\ \left[\mathrm{0,1}\right]\qquad r\ \epsilon\ \left[\mathrm{0,4}\right]$$

Equation (1) is a nonlinear, first-order difference equation. The time-dependent value *x*_*t*_ represents the observation of the variable *x* (population) at time *t*; and the parameter *r* represents the growth parameter that reflects the maximum per capita rate of *x* (Reggiani 2021).

Interestingly, the dynamic Eq. (1) can be considered the first ‘simple’ example of evolutionary complexity in a node/link of a network: it contains only one variable and only one parameter, but it can display stability, cycles, and irregular behaviour, depending on the values of the parameter *r.* In addition, chaos (an irregular domain with unpredictable dynamics) can be interpreted here as a ‘shock’ in a network, depending on high values of the growth rate *r*. In particular, high values of *r,* greater than *r* = 3.824, when the chaos domain occurs, can be related to networks with *fast dynamics* (e.g., financial/internet networks, dynamics of infectious diseases, etc.), as well as to networks with generally *slow dynamics* (such as particular node/links of the traffic systems, physical infrastructure, etc.).

The issue of vulnerability and shock propagation is ‘hidden’ here: Will the stable network be able to stabilise the ‘shock’ of the chaotic node/link/sub-network ‒ or will it be destabilised by this unstable area? The architecture of connectivity, although not explicitly formulated, is certainly important in this (in)stability relationship.

By considering dynamic models of multiple equations, embedding competing/symbiosis or prey-predator relationships, connectivity is ‘*hidden*’ in the interaction parameters. If we examine, for example, the following dynamic system in two dimensions (in discrete time):

2 $$\begin{array}{l}{x}_{t+1}={x}_{t}\left(M-a {x}_{t}-b {y}_{t}\right)\\ {y}_{t+1}={y}_{t}\left(N-c {x}_{t}-d {y}_{t}\right),\end{array}$$

where the coefficients *b* and *c* reflect the interaction between the two dynamic variables *x*_*t*_ and *y*_*t*_,; and *x*_*t*_ and *y*_*t*_ represent, respectively, the values of the variables *x* and *y* at time *t*. *M, N, a*, and *d* are related to the endogenous variable dynamics of each corresponding variable. Since system (2) is expressed in discrete time, unstable and chaotic/unpredictable trajectories may emerge, depending on the values of the parameters and initial conditions (according to the Poincaré-Bendixson theorem) (Reggiani 2021).

However, the structure of connectivity – which strictly impacts vulnerability and chaos movements by means of the interaction parameters *b* and *c* – is not explicitly introduced in the dynamic equations of type (2). It is just ‘captured’ by these two parameters. In addition, if, for example, the first equation in system (2) collapses to an equation of type (1), we model a hierarchical system where the hub, expressed by the evolution of *x*_*t*_, might show chaotic behaviour (through the coefficients *M* and *a*) and influence the evolution of sub-system *y*_*t*_ by means of the *c* coefficient.

In conclusion: the architecture of connectivity has often been ignored in the contributions which concern the spatial economics field. Connectivity configuration is more difficult to detect and measure in spatial economics because it is ‘hidden’ within the (complex) socio-spatial-economic interactions between regions/areas/cities, where the links – and their structures – are less tangible than, for example, in a transport network.

It is worth remembering here all the work on supply-chain modelling as an example of hierarchical systems of network connectivity (Donaghy 2012; Nagurney 2006). Formal considerations on the associated resilience frameworks have been recently highlighted by Donaghy (2022).

On the contrary, connectivity has a distinct, primary role in network analysis, which aims to study complex network representations of physical, biological, and socio-economic phenomena.

## Relevance of Connectivity: Complexity in Network Analysis

*‘Social network analysis (or SNA) involves studying the structure of people's connections‒especially things like who is most important or influential in the network and which groups of people are closely connected*’ (Golbeck 2015, p. 221).

We recall here the fundamental works in SNA by Barabási and his collaborators (see, e.g., Barabási and Albert 1999), who developed new perspectives on spatial-economic-transport networks, by showing that networks often display common behaviour, based on their topological characteristics (see also Newman 2010).

Mostly, topological structures matter according to different types of connectivity (Barabási and Oltvai 2004). The attention to the connectivity issue has given rise, over the years, to a great number of contributions which deal with topology, connectivity, and networks (see, among others, Goyal 2007; Friesz 2007; Naimzada et al. 2009; Reggiani and Nijkamp 2009; Vega Redondo 2007; Vervest et al. 2008). We recall here that the statistical distribution of the links between centres/nodes can be essentially expressed as (Barabási and Oltvai 2004):

- a Poisson Distribution (*random* network);
- a power-law distribution (*scale-free* network).

It should be noted that the statistical connectivity distribution of type a) (random network) identifies a homogeneous socio-economic network, while the statistical connectivity distribution of type b) (Scale-Free/SF networks) identifies the possibility of hubs and a hierarchy of hubs (for a dual economic/network analysis, see Reggiani 2009). Hubs are the preferential nodes/attractors in a network (hub: a single vertex with a large number of connections). According to Barabási and Oltvai (2004), a hub configuration/hierarchy exists for specific values of the *γ* coefficient associated with the power-law distribution. More precisely:

*The smaller the value of γ, the more important the role of the hubs is in the network. Whereas for γ*>*3 the hubs are not relevant, for 2*<*γ*<*3 there is a hierarchy of hubs, with the most connected hub being in contact with a small fraction of all nodes, and for γ* = *2 a hub-and-spoke network emerges, with the largest hub being in contact with a large fraction of all nodes. In general, the unusual properties of scale-free networks are valid only for γ*<*3* (Barabási and Oltvai 2004, p. 102).

These properties are remarkable since they: i) show a very ‘simple’, operational approach for the identification of hubs; ii) paved the way for the use of the various network connectivity indicators, such as network centrality, betweenness, clustering, etc., in order to detect/classify hierarchies of hubs, for example in air transport networks (Alderighi et al. 2007; Burghouwt and Redondi 2013; Reggiani et al. 2010), metro networks (Chopra et al. 2016; Zhang et al. 2019), and transport networks in general (Xie and Levinson 2009).

However, the lack of a formal theory underlying SF networks and their features should be highlighted, as also observed by Li et al. (2005). In this context, it has also been argued that the networks have connectivity that might be stochastic about some underlying mean connectivity represented by a non-normal matrix, and the stochasticity may not be independent and identically distributed across elements of the connectivity matrix (Ahmadian et al. 2015).

A further concern which is worth examining is the issue of the various network topologies vs. the analysis of the related economic weights, We may recall here the fundamental distinction of Simon (1962) between: a) decomposable systems (in which links between subsystems are negligible); b) non-decomposable systems, where links between subsystems are strong and cannot be ignored even in the short run; and c) nearly decomposable systems, in which links within subsystems are strong, whereas links between subsystems are weak but not negligible, so they can be ignored in the short run but not in the long run (Cardinale 2019; Scazzieri 2021). A combination of this connectivity typology by Simon (1962) vs. network topology by Barabási and Oltvai (2004) can be found in Cardinale’s contribution (2019), where the related policy issues are also discussed.

Relevant research issues in this regard are whether the most connected nodes are also the most important ones from the economic viewpoint (or the more ‘open’ to innovation, growth, and mobility):

- Can the current increase of SF networks (Centralised Networks/CNs)) – in this complex globalised world – be considered as an instrument for decoding complexity? (e.g., Google, Amazon, etc.)
- Can a CN of major centres be able to capture the complex dynamics also in terms of vulnerability?

Understanding the (weighted) network topology of a transport/communication system certainly poses some challenges, many of which stem from the identification of the critical/vulnerable structures. Barabási in his recent book (2017) again shows that SF networks/CNs are highly resistant to random failures (a substantial number of links can fail and still not affect the performance of the network as a whole), while they are *very vulnerable to a deliberate attack directed against the major hubs*.

Given these premises, it is worth highlighting a relevant emerging policy issue: Would be it better to design random networks (less ‘vulnerable’ networks?) or more ‘resilient’ SF networks? Undoubtedly, an increase of resilience in the hubs could be the first step towards the design of secure networks, and the architecture of connectivity might help in this regard, as is shown in the following section.

## The Architecture of Connectivity: Resilience in Spatial and Transport Economics

### Towards Network Resilience

Originating from the Latin word ‘*resilio*’, roughly translated as ‘bouncing back’, the idea of resilience means to adapt to or cope with the consequences of (a sudden) change (Rose 2009). The concept of resilience stems from concepts previously developed in the ecological literature, which established two main definitions (Perrings 1998). The first ‘conventional’ definition by Pimm (1984) focuses on the property of systems which are close to some stable equilibrium point (*engineering resilience*). Here, resilience is measured by the speed at which the system returns to equilibrium. The second definition by Holling (1973) focuses on the property of systems which are further away from a stable state. The measure of resilience here is the perturbation that can be absorbed before the system converges on another equilibrium state *(ecological resilience).*

Economic resilience has come to the fore as the ability to absorb the influence of external shocks (Batabyal 1998; Reggiani et al. 2002; Rose 2009, 2015). Economic resilience can analogously be understood as a nation/region/centre/entity’s ability to adapt to (sudden) changes in economic conditions. Based on Martin’s contribution (2012), recent works on (regional) economic resilience tend to describe resilience in a wider sense, including (amongst other things) the abilities to anticipate, prepare, respond to changes, and recover. However, network considerations are not taken into account here.

Three lines of thinking emerge in the analysis of economic resilience (Pendall et al. 2010). Economic resilience is usually studied using a single equilibrium approach, a ‘multiple equilibrium’ approach, or a ‘complex adaptive system’ approach. Studying resilience using a single equilibrium approach means that the socio-economic status of a region ‒ before an external shock ‒ is the equilibrium to which the situation should rebound upon recovery (Pimm’s view (1984)). In a ‘multiple equilibrium’ approach, the system rebounds to a different socio-economic status (Holling’s view (1973, 1992)). In ‘complex adaptive systems’, the notion of shock is replaced with a gradual but constant state of change. Resilience, in this view, becomes the ability to adjust to changes in the total environment of the economy (Christopherson et al. 2010; Fingleton et al. 2012; Pike et al. 2010; Scazzieri 1999; Simmie and Martin 2010).

In summary, resilience has developed into the continuous ability to adjust to stress, and the analysis of resilience has become the study of how the space-economy adjusts to varying stages in economic cycles (Doran and Fingleton 2014; Foster 2007; Pendall et al. 2010). The stages of economic adjustment essentially comprise stages of decline, restructuring, exploitation, and conservation (Dawley et al. 2010; Simmie and Martin 2010).

This latter interpretation has led to a great number of empirical contributions in the spatial economics literature, where different indicators of resilience have been adopted (for a review, see Modica and Reggiani 2015), while neglecting deeper reflections on the theoretical underpinnings of resilience. For example, it would be interesting to analyse whether the structural condition of a system is a prerequisite for identifying resilience. It would seem so, since the first definition of engineering resilience resembles the economic and business cycle concepts (Gandolfo 1996; Samuelson 1939), while the definition of ecological resilience recalls the multiplicity of equilibria advocated by Arthur (1990) and Landesmann and Scazzieri (1990), in the innovation technology field.

From the operational viewpoint an essential issue is the problem of measuring – and thus enhancing – resilience in spatial and transport economics. Operational measures of resilience in a ‘dynamic’ network are not easy to deal with. We might recall here the fundamental work by Rose (2009), in which a series of resilience strategies are provided, such as input substitution, excess capacity, relocation, technological change, etc. In this framework a fundamental role is provided by redundancy. This concept, conceived as untapped or excess economic capacity (e.g., inventories, suppliers), has been considered by Bruneau et al. (2003) as one of the four dimensions of resilience, jointly with: a) robustness (avoidance of direct and indirect economic losses); b) resourcefulness (stabilising measures such as optimising recovery strategies; c) rapidity (optimising time to return to pre-event functional levels) (Rose 2009). In addition, redundancy has been also advocated in network analysis as an important feature of hierarchical organised networks (Burt 1995). However, because of the high costs involved, it is not easy to implement redundancy or additional technologies in the network.

In the spatial economic field, new approaches and socio-economic indicators have recently been taken into account, with reference to resilience at various scale-levels (national, regional and urban) (Alessi et al. 2020; Balland et al. 2015; Pontarollo and Serpieri 2020; Yu et al. 2018). Connectivity or network indicators have rarely been considered in economic analyses of resilience but have recently come to the fore (Boschma 2015; Caschili et al. 2015; Chen and Zhu 2020; Ducruet and Beauguitte 2014; Ferlaino 2020; Yu et al. 2020).

Concerning resilience in transport systems, during the last decade there has been an increase of contributions in this regard (Reggiani et al. 2015; Zanin and Lillo 2013). A recent review on resilience in transportation by Zhou et al. (2019) highlights resilience as the ability to maintain functionality under disruptions, as well as the time and resources required to restore performance level after disruptions. Thus, similarly to what happens elsewhere in the economy, resilience is found to be primarily linked with characteristics such as redundancy, robustness, and adaptation (Gonçalves and Ribeiro 2020).

Topological and network connectivity indicators, which have mostly been used in the past to estimate vulnerability aspects of networks, have recently been utilised in the resilience framework (for a review on resilience in railway transport, see, e.g., Bešinović 2020). Examples of using a quantitative approach for the assessment and improvement of network resilience in any type of network are emerging (e.g., Ahmadian et al. 2020) by also indicating promising avenues from the theoretical viewpoint. These contributions show that the current research interest is moving towards the role of network resilience, and, consequently, towards the various resilience levels and aspects of the fundamental hubs.

### The Architecture of Connectivity: From Network Vulnerability to Resilience

A final issue needs to be mentioned here, i.e., the relationship between the community systems (e.g., travellers, citizens, stakeholders) and the economic/transport network systems, since one system directly affects the other, and vice versa, by contributing to value creation. In other words, the emerging concept of co-creation (Prahalad and Ramaswamy 2004) can also be applied in this context, mostly with reference to the architecture of connectivity between the nodes (and hubs), which can generate some common new properties. For example, the (weighted) connectivity architecture of inter-urban transport networks might originate ‘mobility hubs’ which can also be relevant hubs in the intra-urban transport networks (e.g., a railway station which is also a metro station). This may lead to a discussion of the properties common to hierarchic complex systems, as suggested by Simon (Sect. 1). In particular, as we have seen in Sect. 4, hubs (and hierarchy of hubs) are essential for the formation of SF networks, which can also be nested, given the interrelationships between intra- inter-connectivity networks.

**Fig. 1:**
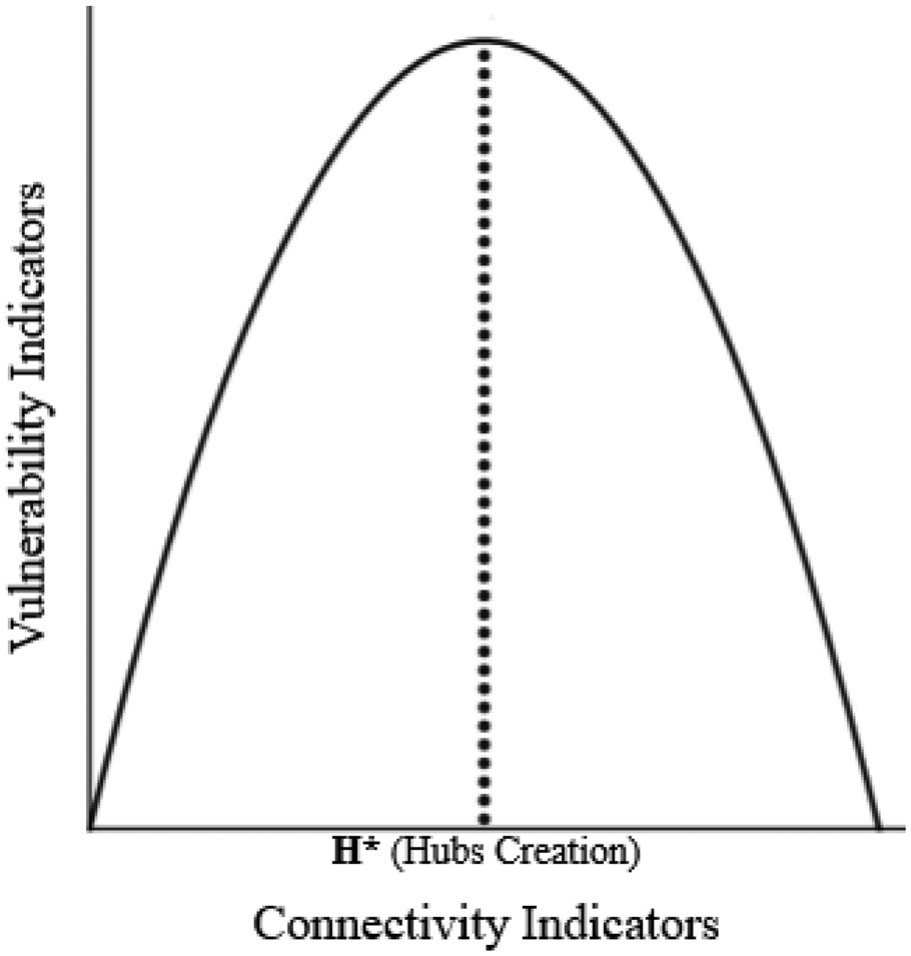
The Vulnerability-Connectivity Curve

Given their high intra- inter-connectivity structures, hubs can be very critical for the network in the presence of shocks; in other words, hubs can be the ‘core’ of network vulnerability. However, hubs are also very resilient, thanks to the ease of communication and interaction in the restoration phase. Hence, with reference to a complex network, we can conceive the vulnerability-connectivity curve as illustrated in Fig. 1.

Figure 1 shows that, in a complex network, connectivity increases in order to build up the *hubs* (point H*), which are, however, also the most *vulnerable nodes* for the network (Sect. 4). We can conjecture here the construction of SF networks. Current research is dealing with different measures of vulnerability and connectivity, depending on the case study under analysis. In general, concerning connectivity, we can assume a composite indicator taking into account a multiplicity of topological network characteristics, such as connectivity degree, centrality, betweenness, etc., usually adopted in complex network analysis (e.g., Boccaletti et al. 2006). Analogously, the choice of the vulnerability indicator depends on the case study under analysis. Network vulnerability can be conceived as a problem of ‘reduced accessibility’, due to disruption (Chen et al. 2007, and Sect. 2.1) and measured by a (composite) measure of accessibility (Taylor et al. 2006). Alternatively, network vulnerability can be measured by link vulnerability indicators (Knoop et al. 2012; Neves et al. 2021), or by network efficiency/performance indicators (Nagurney and Qiang 2007).

In particular, in Fig. 1, we assume an inverted-U-shape relationship between vulnerability and connectivity indicators, which recalls the Environmental Kuznets Curve between different pollutants and per capita income (Dinda 2004; Kuznets 1955). In Fig. 1, the maximum value of the vulnerability curve coincides with the creation of hubs (or hierarchy of hubs) leading to an SF network. Above this value H* (Hubs creation), connectivity can be an instrument enhancing resilience, which is, in turn, a way to reduce vulnerability.

To sum up, connectivity certainly plays a fundamental role in complex spatial economic networks, by creating various network topologies (e.g., hub-and-spoke networks, hierarchical networks, SF networks, etc.). In particular, the latest findings in network theory show how – for certain network configurations – distant things can be related by means of ‘hubs’ or ‘egos’ (preferential nodes/attractors). The architecture of hubs/hierarchy of hubs can shed new light on the emerging vulnerability/resilience patterns (Albert et al. 2000; Barabási 2017; Cardinale 2019; Lichter et al. 2022; Reggiani 2013; Scazzieri 2021).

In addition, the architecture of connectivity seems to play a fundamental role in what is called ‘*creative destruction’*: vulnerability effects associated with chaos models/properties (Sect. 3.2) might be revisited in a positive perspective, by means of ecological resilience (movements towards new equilibria). In other words, small changes, which can provoke high unstable/uncertain/chaos effects in the hubs, are not necessarily negative, thanks to network connectivity leading to resilience (Fig. 1).

The new equilibria – even though arising from the destruction of the previous ones – can create new opportunities by recalling the ‘old concepts’ from Greek mythology: Chaos as the divinity (Socrates) or as disordered motion before the divine order (Plato) (McCabe 1997).

**Fig. 2:**
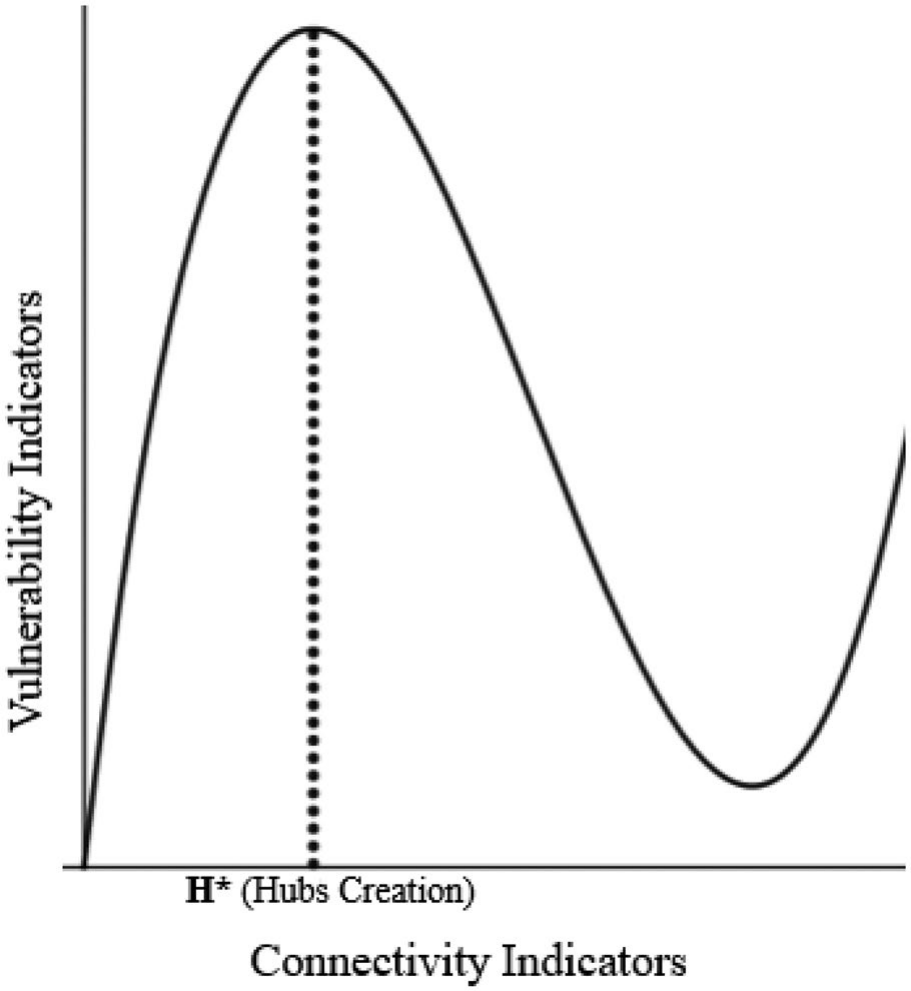
Possible Evolution of the Vulnerability-Connectivity Curve

In general, the architecture of connectivity in a globalised network can be conceived as the conductive platform for further spatio-temporal vulnerability domains. In this long-term scenario, the inverted U-shaped curve in Fig. 1 might be substituted by an N-shape, showing alternate periods of high-low–high vulnerability which also depend on the network connectivity configuration (Fig. 2).

Advanced theoretical and empirical studies are certainly necessary in this respect.

## Conclusions

‘*In its simplest form, connectivity is a description of the level of connectedness within a system, and corresponds to a structured set of relationships between spatially and/or temporally distinct entities* (Kool et al. 2013)’ (Turnbull et al. 2018, p.1).

Connectivity, conceived of as the capacity/ability to make and maintain a connection between two or more points in a spatial network, appears to be central in network and spatial economic science, while the role of its architecture needs more in-depth attention from both the theoretical and operational viewpoint. The present paper has aimed to review and analyse the concept of connectivity, in the light of its network structure and its related impact on vulnerability, complexity, and resilience.

It appears that the architecture of connectivity is fundamental to understanding and interpreting – in the (spatial-) economic networks – vulnerability and resilience. In particular, a (weighted) network connectivity may:

- Identify different typologies of hubs/hierarchies of hubs in a complex network, and, consequently,
- Identify the associated vulnerability and resilience patterns in the ‘influential’ areas of the network.

Therefore, a research agenda in this regard needs to focus on the different connectivity network structures leading to the various types of hubs, in parallel with the research on the related hubs’ measures of vulnerability and resilience.

In terms of (dynamic) theory, more work could be undertaken, starting, for example, from Gao et al. (2016) and Vespignani (2010). In terms of measurement and operationalisation, the identification of the architecture of hubs is not easy, given the various connectivity indicators and measures, but it could lead to novel developments.

From the policy viewpoint, a central issue is the tendency towards a network-centric organisation, which involves the cost of transforming socio-economic-transport random networks into SF networks, and consequently the possibility of incurring considerable vulnerability in areas dominated by hubs. Decision strategies that strongly influence the topology and dynamics of complex networks – and thus the emergence/death of hubs – might consider the impact of connectivity architecture, not only on vulnerability, but also on resilience. In addition, the inter-connectivity of hubs is a cause of potential vulnerability/resilience which should be taken into account.

In this research endeavour, ‘Scientia’, which involves the development of theories, analyses, and experiments/applications in an interdisciplinary framework, is vital. Hence, a multidisciplinary perspective on the architecture of connectivity is a challenge for properly understanding network complexity.

A research platform in this regard is certainly provided by complexity science, which can offer a comprehensive and common umbrella, by synthesising and interpreting different concepts, theories and tools for investigation, as well as by showing its practical value for society, as argued by Johnson (2010, p. 132): …*the emerging science of complex systems will embrace the humanities and conventional social sciences in the same way that it has embraced concepts from the natural sciences. This will involve a synthesis of knowledge from the different scientific traditions, and a synthesis of those traditions into a new science applicable at all metalevels of human affairs.*

## Data Availability

Data sharing not applicable to this article as no datasets were generated or analyzed during the current study.

## Footnotes

1 Spatial economics encompasses quite a broad range of literature, from the ‘new economic geography’ (Fujita and Thisse 2002) to the ‘super-networks’ analyses (Nagurney and Wakolbinger 2005), in which connectivity plays a relevant role (for a review, see Donaghy 2021). Given the breadth of this literature in this regard, in this paper we aim to pay particular attention to the specific issue of the architecture of connectivity.
